# *In Vitro* Model-Systems to Understand the Biology and Clinical Significance of Circulating Tumor Cell Clusters

**DOI:** 10.3389/fonc.2018.00063

**Published:** 2018-03-12

**Authors:** Alexander N. May, Bryan D. Crawford, Aurora M. Nedelcu

**Affiliations:** ^1^Biology Department, University of New Brunswick, Fredericton, NB, Canada

**Keywords:** cancer, metastasis, circulating tumor cell clusters, circulating tumor cell, E-cadherin, vimentin, desmosomes

## Abstract

The isolation of clusters of circulating tumor cells (CTCs) from cancer patients has recently challenged the accepted view that the initiation of secondary tumors during metastasis involves the dissemination of individual cancer cells. As such clusters appear to be more aggressive than single tumor cells, CTC clusters are now considered a main player in the metastatic process, and many studies are exploring their diagnostic, prognostic, and clinical significance. However, several technical challenges limit advances in this area. Here, we suggest the use of established cancer cell lines that grow as cell clusters in suspension as a complementary approach that can help in understanding the biology of CTC clusters and their clinical significance. We argue that the many similarities between these “surrogate” clusters and the CTC clusters isolated from patients (e.g., in terms of size, morphology, heterogeneous expression of epithelial and mesenchymal markers, and type of cell–cell junctions) make these cell lines ideal systems for the development of strategies aimed at preventing or slowing down the metastatic process by targeting CTC clusters.

## Introduction

Metastasis is responsible for most cancer-related deaths (1, 2). Until recently, the initiation of secondary tumors was thought to involve the dissemination of individual cancer cells. Consistent with this view, single circulating tumor cells (CTCs) have been found in the blood of cancer patients [reviewed in Ref. (3)]. However, more recently, the isolation of *clusters* of CTCs from blood samples has challenged this paradigm, as such clusters appear to be more aggressive than single CTCs (4, 5) and increase in frequency during metastasis (6). CTC clusters are now considered a main player in the metastatic process, and the current focus is on exploring their diagnostic, prognostic, and clinical significance (7). However, research in this area is hindered by several technical challenges (discussed below). Here, we suggest a complementary approach poised to help our understanding of the biology of CTC clusters and the development of strategies aimed at preventing or slowing down metastasis by targeting CTC clusters.

Circulating tumor cell clusters isolated from blood samples range from 2 to 100 cells (with most clusters comprising between 20 and 40 cells), and may also contain cancer-associated fibroblasts, platelets, and immune cells (hence their being also referred to as circulating tumor microemboli) (8). Cells in a cluster maintain strong cell–cell connections, including desmosomes and adherens junctions, the presence of which is thought to confer resistance to anoikis (7, 9–12). Consistent with the presence of cell junctions, CTC clusters are comprised mostly of cells with epithelial characteristics (and expressing epithelial markers—such as EpCAM, E-cadherin); but cells with mesenchymal markers (e.g., vimentin) or a combination of both epithelial and mesenchymal features are also observed (12–14), indicating substantial cell heterogeneity and/or plasticity within a cluster. The presence of both cell types and/or the ability to shift between states is thought to contribute to their higher metastatic potential relative to single CTCs, while the absence of proliferation markers (15) might explain their higher resistance to chemotherapy (14, 16). A link between the size of CTC clusters and patient’s overall survival has also been made recently, with larger-size CTC clusters conferring a higher risk of death (17).

## The Problem

Despite the accepted view that CTC clusters are extremely relevant to the metastatic process and that their presence correlates with poor clinical outcome, research on the potential of CTC clusters as therapeutic targets is hampered by several factors. For instance, blood samples contain significantly fewer CTC clusters than single CTCs [ca. 2–5 vs 95–98% (4); but see Ref. (6) for higher estimates]. Athough several isolation techniques for CTCs have been recently developed, the available methodology for the isolation of CTC clusters (e.g., immobilization of clusters onto micropillars; microfluidic chips that use deterministic lateral displacement to sort clusters based on size and asymmetry) is still difficult, inefficient, and can result in the deformation and dissociation of cell–cell contacts (7, 18, 19). Furthermore, although *ex vivo* culturing of single CTCs is now possible [e.g., Ref. (4, 20–25)], the propagation of CTC clusters is rather challenging and requires hypoxic and pressurized conditions as well as serum-free media supplemented with growth factors (10, 26, 27). Last, cultured CTCs can eventually assemble in large spheroids (up to 1 mm)—known as “tumorospheres” (20, 21, 23, 27), which do not accurately represent the biology of CTC clusters.

Given these difficulties, alternative systems have been employed to “mimic” the CTC cluster phenotype. For instance, cell clumps formed during the trypsinization of adherent cell lines or during their growth in non-adherent conditions have been used to investigate the metastatic potential of CTC clusters (4, 5), their increased resistance to drugs (16), and their ability to traverse capillary sized vessels (26). Nevertheless, the similarities between these clumps and CTC clusters (with respect to cell–cell contacts and expression of CTC-specific markers) have not been fully addressed. Likewise, complex methods for the development of multicellular and single cell-derived tumor spheroids from established pancreatic cell lines (using hanging drop and ultra-low attachment plates) have been recently reported as means to mimic the growth pattern of CTC clusters (28), but the size and morphology of these spheroids (large compact or hollow spheres) have little similarities to isolated CTC clusters.

## *In Vitro* Model-Systems to Investigate the Biology of CTC Clusters

Here, we suggest a complementary approach to explore the biology and potential therapeutic value of CTC clusters. Specifically, we propose that presently available established cell lines that grow as cell clusters in suspension can be used as “surrogates” for CTC clusters. The main similarities between these clusters and the real CTC clusters, include: cluster size and morphology, expression of CTC-specific markers, cell heterogeneity and plasticity, and type of cell–cell connections.

**Figure 1:**
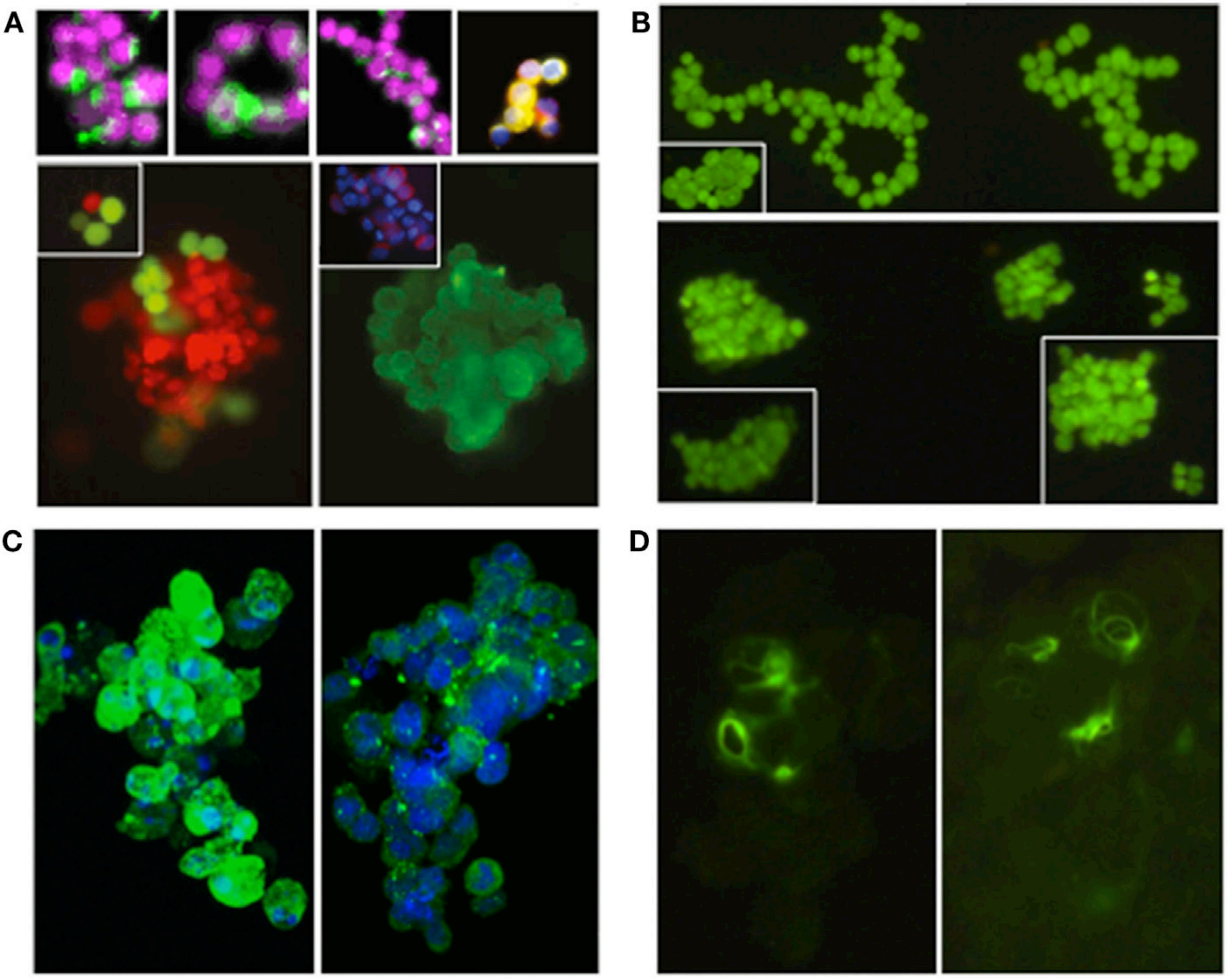
Real circulating tumor cells (CTC) clusters from patients with various types of cancer and “surrogate” CTC clusters from two established lung cancer cell lines (NCI-H187 and NCI-H2122). **(A)** CTC clusters isolated from individuals with small cell lung cancer [top row—left and middle panels (14)], prostate cancer [top row—right panel (4)], and breast cancer [bottom row (4)]; for staining details see references (images reproduced with permission from publishers*). CTC clusters from Ref. (14) were isolated using the CellSearch CTC Kit (Veridex, Warren, NJ, USA)—based on the co-expression of EpCam and CK-8, -18, and -19 markers, and the exclusion of cells expressing the white blood cell marker CD45 (29), as well as a membrane filtration system using the ISET platform (Metagenex, Paris, France) (30). CTC clusters from Ref. (4) were isolated using the HBCTC-Chip system (which employs biotinylated antibodies against EpCAM, EGFR, and HER2 to trap CTCs in microfluidic chambers) (12, 31) and the negCTC-iChip system (based on the depletion of both leukocytes and erythrocytes, followed by the identification of remaining CTCs) (32). **(B)** Cell clusters from H2122 (top row) and H187 (bottom row) cell lines (stained with Syto-9). **(C)** Cell clusters from H2122 (left panel) and H187 (right panel) immunostained for E-cadherin (in green; nuclei stained with DRAQ5 in blue); non-membrane localization of E-cadherin was also observed in real CTC clusters (14). **(D)** Cell clusters from H187 immunostained for vimentin (in green); vimentin is also heterogeneously expressed in real CTC clusters (14). *Images reprinted from Ref. (4), Copyright (2014), with permission from Elsevier. Images reprinted from Ref. (14), Copyright (2011), with permission from Elsevier.

**Figure 2:**
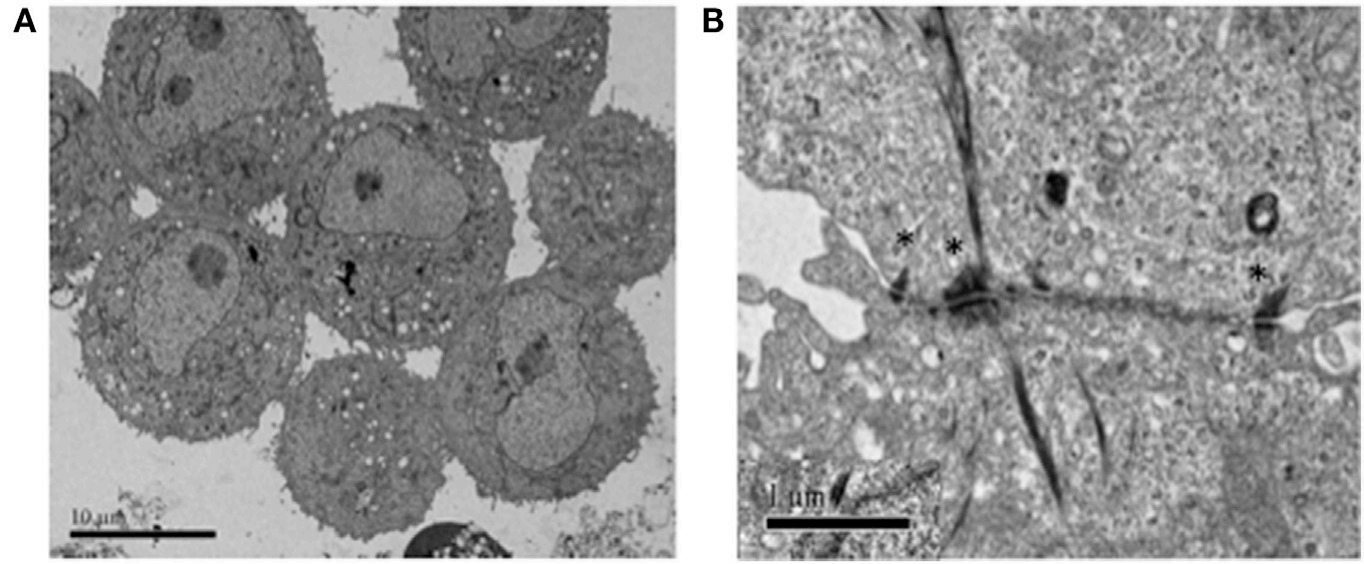
Electron microscopy showing the types of cell–cell connections between cells in H2122 cell clusters. **(A)** Extensive and complex connections among cells in a cluster, including both loose microvilli interactions as well as structured cell–cell contacts. **(B)** Cell–cell junctions involving desmosomes (denoted by *) between two cells in a H2122 cell cluster.

In Figure 1, we present two lung cancer cell lines (obtained from the American Type Culture Collection; https://www.atcc.org/) as examples and proof-of-principle. NCI-H187 is a small cell lung cancer line (derived from cells recovered from pleural effusion, prior to therapy) that grows as multicellular aggregates in suspension. NCI-H2122 is a non-small cell lung cancer line (also established from metastatic pleural effusion) that shows a mixture of both adherent cells and grape-like cell clusters in suspension. Under typical growth conditions, the two cell lines form clusters that are similar in size and conformation to CTC clusters isolated from patients with various cancer types—including lung, breast, and prostate cancer (Figures 1A,B). Also, as shown for real CTC clusters, these surrogate CTC clusters express both epithelial and mesenchymal markers (i.e., E-cadherin and vimentin, respectively; Figures 1C,D), indicating substantial cell heterogeneity and/or plasticity within a cluster. Such features are thought to contribute to the collective migration and higher metastatic potential of CTC clusters relative to single CTCs [e.g., Ref. (7, 8, 14)]. Furthermore, consistent with the presence of E-cadherin, electron microscopy reveals that cells in these clusters are held together by adherens junctions and desmosomes (Figures 2A,B), which are features that have been suggested to contribute the CTC clusters’ resistance to anoikis (4).

Based on these similarities, we argue that established cell lines that grow as cell clusters in suspension provide a series of advantages that have not been explored and exploited. Due to their ease of culturing and analysis, many general aspects that are relevant to real CTC clusters can be investigated in these cell lines. For instance, one therapeutic strategy that has been proposed to decrease the metastatic potential of CTC clusters is to dissociate them into single cells. Experiments using agents that can interfere with cell-cell adhesion showed promise [e.g., inhibiting the synthesis of plakoglobin, which is a protein found in desmosomes and adherens junctions—including those in CTC clusters (33)] (4, 5). However, the generation of candidate molecules to interfere with cell–cell contact mechanisms requires both the development of new high-throughput screening endpoints and the availability of a large number of CTC clusters (9). Since isolation techniques as well as *in vitro* culturing of isolated CTC clusters are still challenging, cancer cell lines that grow as clusters in suspension can facilitate the development of such methodologies as cells in these clusters exhibit the same adhesion mechanisms as cells in CTC clusters (Figure 2). Furthermore, experimental evolution can be used in other cell lineages of interest to select for growth in suspension; using this approach we were able to select for an NCI-H2122-derived cell line that grows mainly in suspension (our unpublished data). The availability of cell clusters independently evolved from various cell types and on distinct genetic backgrounds will allow for the identification of common markers and properties (including new proteins potentially important for the survival and increased metastatic potential of CTC clusters) that can be exploited for new therapeutic strategies.

Additionally, such cell lines can be used to investigate the mechanisms that confer increased chemo- and radio-resistance upon CTC clusters (e.g., integrin signaling, cell–cell contact, cell progression blocks). Drug-resistance can also be selected for in these lines, and new therapeutic approaches that can decrease the survival of resistant CTC clusters or interfere with their potential to establish new metastases can be tested. Cell lines with various genetic backgrounds and mutation panels are available, which can expand the range of studies addressing the effect of general or specific/targeted drugs on CTC clusters.

Although the surrogate CTC clusters cannot provide insights into the first phase of the metastatic process (the physical dissemination of tumor cells), they can facilitate our understanding of the “colonization” phase—a process that has been identified as requiring additional research (22). For instance, these CTC clusters can be xenografted in mouse or other models and their metastatic potential traced, evaluated, and experimentally manipulated. Last, cell lines that grow as a mixture of both adherent and suspension cells can be used to examine differential responses to drugs between the two phenotypes (solid tumors and CTC clusters, respectively) as well as investigate conditions that favor the adherent phenotype over the suspension phenotype [such as an increase in pH; (34)], and thus potentially lower the disseminating potential of tumors.

## Conclusion

In this perspective, we suggest that established or experimentally evolved cell lines that grow as cell clusters in suspension can improve our understanding of the biology of real *in vivo* CTC clusters and facilitate the development of strategies aimed at preventing or slowing down metastasis by targeting CTC clusters. We do recognize that there are limitations associated with the use of these “surrogate” CTC clusters, including the absence of non-cancer cells, the presence of which is thought to influence the properties and responses to therapy of real CTC clusters. However, we believe that the accessibility and affordability of such cell lines for both *in vitro* and *in vivo* studies and the potential for standardizing and replicating such studies (allowing for meta-analyses) are features that can complement the more limited studies can be performed currently with isolated primary CTC clusters.

## Author Contributions

AM, BC, and AN designed the study, interpreted the data, and wrote the manuscript. AM and BC carried out the experiments.

## Conflict of Interest Statement

The authors declare that the research was conducted in the absence of any commercial or financial relationships that could be construed as a potential conflict of interest.
